# Network Centrality of Metro Systems

**DOI:** 10.1371/journal.pone.0040575

**Published:** 2012-07-06

**Authors:** Sybil Derrible

**Affiliations:** Future Urban Mobility Inter-Disciplinary Group, Singapore-MIT Alliance for Research and Technology, Singapore, Singapore; Umeå University, Sweden

## Abstract

Whilst being hailed as the remedy to the world’s ills, cities will need to adapt in the 21^st^ century. In particular, the role of public transport is likely to increase significantly, and new methods and technics to better plan transit systems are in dire need. This paper examines one fundamental aspect of transit: network centrality. By applying the notion of betweenness centrality to 28 worldwide metro systems, the main goal of this paper is to study the emergence of global trends in the evolution of centrality with network size and examine several individual systems in more detail. Betweenness was notably found to consistently become more evenly distributed with size (i.e. no “winner takes all”) unlike other complex network properties. Two distinct regimes were also observed that are representative of their structure. Moreover, the share of betweenness was found to decrease in a power law with size (with exponent 1 for the average node), but the share of most central nodes decreases much slower than least central nodes (0.87 vs. 2.48). Finally the betweenness of individual stations in several systems were examined, which can be useful to locate stations where passengers can be redistributed to relieve pressure from overcrowded stations. Overall, this study offers significant insights that can help planners in their task to design the systems of tomorrow, and similar undertakings can easily be imagined to other urban infrastructure systems (e.g., electricity grid, water/wastewater system, etc.) to develop more sustainable cities.

## Introduction

The advent of cities as one-fit-all solutions in the 21^st^ century is unequivocal. Hailed as the answer to the demographic problem (as popularized by the 7 billion series by National Geographic [1]), and the remedy to all ills (in the form of the City 2.0 as dubbed and prized by TED [2]), cities represent all that works for a sustainable and resilient future. All over the world, myriad researchers have decided to focus their efforts on studying cities, and a cornucopia of patterns, findings and properties have been uncovered [3]–[5]. One discipline that seems to particularly stand out is *complexity*: cities as complex systems [6]–[12]. As self-organizing, evolutionary, and highly competitive environments, cities are indeed complex akin to countless systems in the universe.

One dominant aspect of cities is transportation. Indeed, the transport system is essentially the lifeblood of cities. Through the movement of people and goods, the transport system is a significant factor influencing (both negatively and positively) economic activity [13], [14], social development [15], [16], public health [17] and livability [18], [19]. More specifically, the network feature of transportation presents great opportunities, which can be analyzed from the viewpoint of network science [20], [21]. Many researchers have tried and succeeded in adopting a complex network approach to study cities’ transportation systems [22]–[27]. More specifically, public transportation carries special relevance since it is often considered as the main competitor to the private automobile for a sustainable future. As a result, public transport systems are likely to grow significantly in the future, and they need to be able to accommodate the growing urban population, which is a colossal challenge. Recent gasoline price surges have produced a noticeable increase in transit ridership, and it appears that current systems are undergoing a lot of stress and are in no way, shape or form able to cope with a substantial increase of riders [28], [29]. Traditional transit planning technics must therefore be updated and adapted to be able to address this problem, and here again taking a complex network approach may be beneficial.

**Figure 1:**
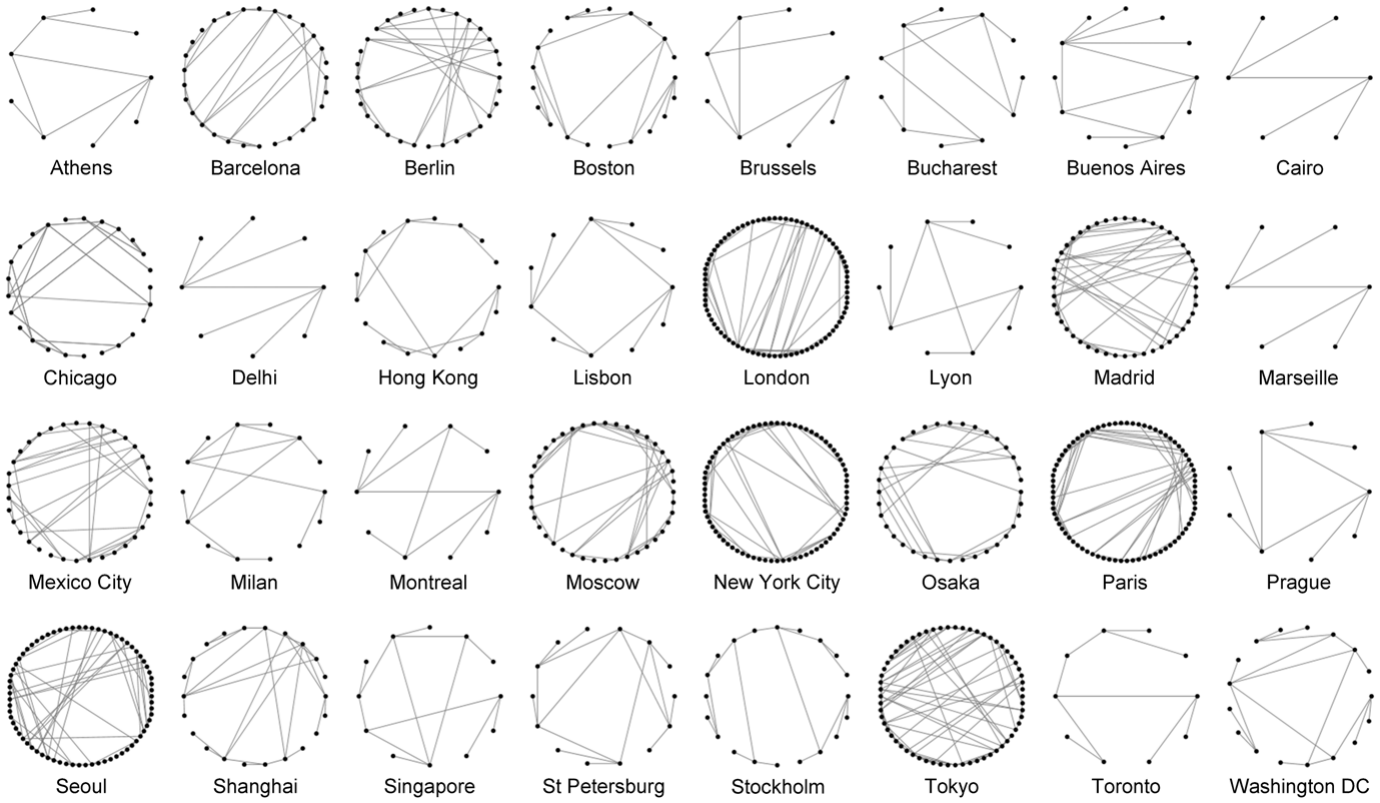
Circle representation of 32 metro networks in the world (using NodeXL [**52**]).

Several studies have already looked at public transport systems as complex networks, and many relevant properties have been discovered, including scale-free and small-world features [30]–[35]. This is somewhat surprising considering transit systems are designed to fit the needs of specific regions and global patterns are not necessarily intuitive. Moreover, these patterns and properties can actually have an impact on ridership itself [36], [37]. Looking at transit systems as complex networks can therefore have many benefits, one of which is to enable a holistic view of the system. For instance, Figure 1, which clearly echoes Watts and Strogatz famous figure [38], offers a novel way to view and analyze metro networks. One advantage of transit systems is their relative smaller size compared to most studied networks, which makes an initial visual inspection possible. The main goal of this paper is scrutinize one of the most important features of transit systems: network centrality.

Network centrality is a fairly old concept that emerged in the 1950s from social sciences [39]–[41]. Although it has been fairly extensively studied for larger complex networks [42], work applied to transit seems relatively scarcer. For transportation in general, the use of network centrality has been mostly applied to study matters of robustness [32], and it is slightly more common in transport geography, notably to study the relationship between transport and land-use [43]–[45]. Better understanding the evolution of centrality in transit systems has clear benefits; one practical application is determine what stations in the system are more central and thus design the system so as to distribute the flow of passengers more evenly; another application is to develop a tool to forecast ridership increase linked with the opening of new lines. Many centrality indicators have been developed in the past, from the simple degree centrality (i.e. number of connections per node) to the PageRank indicator used by Google [46]. For this work, most existing indicators were considered but betweenness centrality clearly stood out as showing particular relevance and an interesting behavior. Unlike the other indicators, betweenness highlights the importance of a node as a transfer point between any pairs of nodes. This *transfer* characteristic is obviously of paramount importance in transit systems. Moreover, betweenness offers a pragmatic way to capture the urban context (i.e. geographic factors), making the location of a station a key feature (unlike degree centrality for instance that only counts the number of connections).

By scrupulously analyzing the properties and effects of betweenness of 28 metro systems in the world, the objectives of this work are to (1) briefly present the methodology to collect data and calculate betweenness, (2) study the emergence of global trends in the evolution of betweenness, (3) analyze the impact of betweenness by looking at individual stations of a few selected systems. Metro here refers to urban rail transit with exclusive right-of-way, whether underground, at grade, or elevated, often colloquially referred to as metro, subway, underground, tube, etc. The choice of metros was natural since they are essentially closed systems, not constrained to follow road patterns, and often representative of their cities.

As we will discover, betweenness behaves in interesting ways in metro systems, sometimes symptomatic of city-wide conditions (i.e. travel patterns), but a methodology to effectively measure betweenness must first be presented.

## Methods

### Collecting Data

Network centrality, in this paper, is a topological property (i.e. related to the geometry of the network and not the flows). The first step towards analyzing centrality is therefore to collect data about its structure. This is typically done by representing the network as a graph *G* with *N* nodes/vertices and *M* links/edges. By having stations/stops all linked by lines, public transport systems are in fact physical networks. Nevertheless, there exists several ways to define them as graphs; see [47].

In this paper, only the termini and transfer stations are taken as nodes, other stations that do not offer transfers or that do not end lines are simply not considered. By having lines, metros vary more typical networks, and studying them is not trivial (let alone add other transit modes [34]). The rationale behind this decision is to focus on the transferring properties of metros; in other words, learning that a non-transfer station in the middle of a line is most central does not necessarily offer helpful information. For further information about the methodology, see [48].

**Figure 2:**
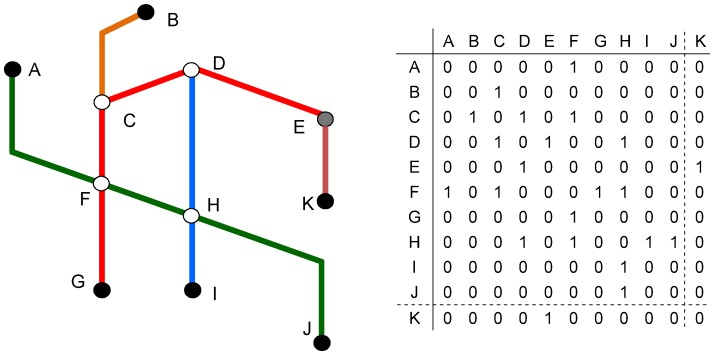
Schematic graph of Lyon metro system and its adjacency matrix. The left side is the sketch of the system where the shapes of the lines are kept even though the graph is isomorphic. Termini are illustrated by black circles and transfers stations by white circles. The right side of the graph shows the adjacency matrix (i.e. ‘1′ when a connection exist and ‘0′ otherwise). Note that link EK does not exist in real life, hence the greyed node E and the dotted line in the matrix.

As an example, Figure 2 represents a sketch of the Lyon metro. The metro has a total of four lines and 39 stations, but only the six termini (black circles) and four transfer stations (white circles) are considered nodes. Note that the link EK does not exist in reality (thus not counted here), it is only added to show evolution in betweenness later on, hence the greyed shade (acting both as a terminal and transfer station). The figure also contains the adjacency matrix of the Lyon metro; i.e. cells have a value of ‘1′ if a connection exists, and ‘0′ otherwise.

Two points should be further noted. First, unlike most networks, transit systems have lines, and therefore riders do not have to transfer at each transfer station. Although this is a significant property, somewhat unique to transit, it should not affect a general study on network centrality. Second, multiple links are not included in this analysis; i.e. multiple lines connecting two consecutive stations. For instance, the addition of links to connect nodes G to F and F to C in Figure 2 would not alter the adjacency matrix since these new links only offer already existing connections (i.e. redundant information). To take a more practical example, the station République in the Paris metro hosts five non-terminating lines, which would suggest at first that the station has a number of 10 connections (i.e. 10 links connected to one node). However, six of these 10 connections link two lines to the same pair of stations (e.g., lines 5 and 9 both connect République to Oberkampf), which reduces the number of connections to 7. This technicality can carry some impact, in particular for degree centrality (not studied here).

### Defining Betweenness Centrality

The concept of betweenness centrality was first introduced by Freeman [49] in the 1970s to study social networks. The logic behind betweenness differs from most centrality indicators. Indeed, the importance does not rely so much on the location of the node as an end point, but on whether or not it is used to join any two other nodes (taking the shortest paths). This is particularly relevant in the case of public transport. A station might be heavily used because it is in the vicinity of an important location (e.g., central business district, entertainment area, etc.), but another station may be even more heavily used because it serves as a transfer point to get to many locations. For instance in Lyon (Figure 2), node F services the Place Bellecour (major shopping center) and it is heavily used. Meanwhile, although not located in a major area, node H is heavily used simply to get to node F and to stations towards node D to reach the Part-Dieu area (also a major shopping area, as well as the financial district and the main train station).

Mathematically, each node is first given a probability by counting how many times it is used to link any pair of nodes. For example in Figure 2, there are two possible shortest paths to go from node C to H, one path by going through node D and the other through node F. The probability to use node D is therefore ½, and similarly for node F. More generally, let *p_jk_* be the total number of shortest paths linking nodes *j* and *k*, and *p_jk_(i)* be the number of these paths going through node *i*, then the probability of using node *i* is simply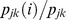. Doing this for all node pairs, the mathematical formulation of betweenness centrality is:
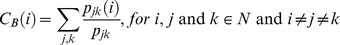
(1)


To achieve high betweenness, the numerator should be as high as possible; hence nodes with high values of *C_B_* are considered more central. Because larger networks have more possible paths, betweenness systematically increases with network size. It can therefore be desirable to standardize the results. Freeman [49] suggested dividing the betweenness centrality of each node by the maximum possible betweenness centrality for a graph with |*N*| nodes, which is 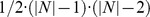 for a star network. This process, however, simply results in inversing the previous trend (i.e. now betweenness systematically decreases with size), and it is therefore not necessarily helpful in our case. For this work, it is preferable to simply normalize the results by dividing the betweenness of each node by the sum of all nodes (equation 2), thus binding betweenness between 0 and 1.
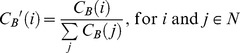
(2)


Normalized betweenness notably enables us to compare cumulative distribution of betweenness in metros as will be shown in the next section.

In Lyon, betweenness centralities are C (11, 0.19), D (11, 0.19), F (18, 0.31), H (18, 0.31), for the current network (i.e. no link EK), where information in brackets are original and normalized centralities respectively. Here, nodes F and H are equally central, followed by nodes C and D. Note that termini do not have any betweenness since they can never be on the pathway between two other nodes.

In practice, there are various ways to calculate these indicators from adjacency matrices. Because shortest-paths have to be calculated, algorithms such as the Floyd algorithm and the Dijkstra algorithm [50], [51] can be used. In this paper, the free open-source Microsoft Excel 2007/2010 add-in NodeXL [52] was used.

## Results

The methodology described was applied to 28 metro systems in the world. Although there are arguably more than 150 metros in the world, most of them are small or merged with other transit modes (e.g., light rail transit), and the purpose here was to gather a representative pool of systems; Ovenden’s *Transit Maps of the World* [53] was used for the selection. Table 1 presents the data collected and calculated for these 28 metro systems, ordered from smallest to largest in terms of number of nodes. It first contains the number of nodes and links per system, followed by the values of betweenness centrality calculated for each system, where Min, Max, Ave, and Sum stand for minimum measured (non-zero values since termini necessarily have zero betweenness), maximum measured, average calculated, and sum of all betweenness (for the normalization) respectively. A third column entitled “Quadratic Coefficients” is also present, where subscripts ‘*n*’ and ‘*o*’ stand for ‘normalized’ and ‘original’ respectively; they will be explained later on.

**Table 1:** Results for 28 metro networks.

Metro	Nodes	Links	Betweenness Centrality *C_B_*				Quadratic Coefficients	
			Min*	Max	Ave	Sum	|a_n_|	|a_o_|
Athens	9	9	7.00	15.00	5.33	48	0.03125	1.50
Brussels	9	9	7.00	19.00	5.11	46	0.03261	1.50
Lyon	10	10	11.00	18.00	5.80	58	0.03017	1.75
Montreal	10	10	11.00	18.00	5.80	58	0.01742	1.75
Toronto	10	9	8.00	26.00	8.20	82	0.03017	1.43
Bucharest	11	12	6.00	19.00	6.82	75	0.01643	1.23
Lisbon	11	11	13.50	21.50	6.82	75	0.02167	1.63
Singapore	12	13	10.00	26.00	8.92	107	0.01335	1.43
Buenos Aires	12	13	17.50	34.00	7.33	88	0.00426	0.38
Milan	14	15	23.00	39.00	12.36	173	0.00888	1.54
St Petersburg	14	16	22.50	25.00	9.93	139	0.00058	0.08
Hong-Kong	17	18	15.00	71.00	18.94	322	0.01046	3.37
Washington DC	17	18	19.00	71.00	16.94	288	0.00544	1.57
Stockholm	20	19	35.00	113.00	28.60	572	0.00920	5.27
Boston	21	22	37.00	102.00	25.62	538	0.00592	3.18
Shanghai	22	28	13.83	93.22	21.82	480	0.00441	2.12
Chicago	25	57	23.00	221.00	63.92	1598	0.00397	6.34
Barcelona	29	42	9.12	163.07	35.45	1028	0.00218	2.25
Berlin	32	43	22.10	110.40	40.97	1311	0.00166	2.17
Mexico City	35	52	12.42	129.22	44.20	1547	0.00111	2.06
Osaka	36	51	13.25	153.00	50.25	1809	0.00133	2.01
Moscow	41	62	25.52	177.26	54.10	2218	0.00106	2.36
Madrid	48	79	2.03	265.19	72.77	3493	0.00062	2.18
Tokyo	62	107	9.50	452.55	98.56	6111	0.00045	2.76
Seoul	71	111	21.41	467.57	144.10	10231	0.00034	3.50
New York City	77	109	10.74	683.15	162.45	12509	0.00036	4.46
Paris	78	125	40.07	630.73	152.50	11895	0.00025	3.02
London	83	121	7.69	1240.29	185.84	15425	0.00030	4.59

* minimum non-zero values since termini have no betweenness.

Data for the metro systems were collected in 2008–2009, and discrepancies to current systems might exist (in particular for the Shanghai metro that has substantially increased).

### Global Trends

**Figure 3:**
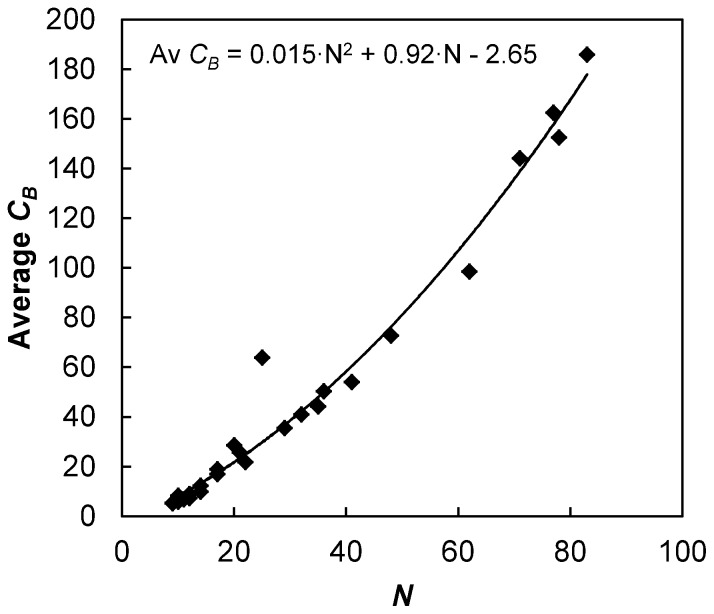
Evolution of average betweenness centrality *C_B_* with network size. The regression fits a second degree polynomial and the statistical significance is surprisingly high; only Chicago does not fit the regression as well (perhaps due to its five-lined directed elevated section in the so called “loop” area).

Conceptually, the relevance of betweenness to public transport systems is fairly intuitive. From Table 1, the system with the lowest average is Brussels (one of the smallest metros) and the system with the highest average is London (the biggest system). It is clear that average betweenness tends to increase with size, which is natural considering the definition of betweenness (i.e. betweenness increases with the total number of shortest-paths). Nevertheless, the statistical significance of the regression is surprisingly high (Figure 3) and the fit follows a second degree polynomial of equation 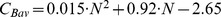. The quadratic nature is interesting and perhaps related to the planarity of metro networks (i.e. two links crossing each other systematically creates a new node). Indeed, the number of nodes is a one-dimensional parameter and betweenness centrality in planar networks is a two-dimensional parameter (although further efforts would be needed to generalize the trend). Moreover, it essentially means that the rate of increase of shortest-paths grows faster with size, and it increases linearly (although fairly slowly with a coefficient of 0.015). Note that the one metro that does not fit the regression is Chicago (25 nodes), which is due to the presence of the so-called “loop” in the downtown area that hosts five lines, increasing substantially the number of links and in turn the number of paths; despite having a similar number of nodes, Chicago has twice the number of links as Shanghai, hence the higher betweenness.

**Figure 4:**
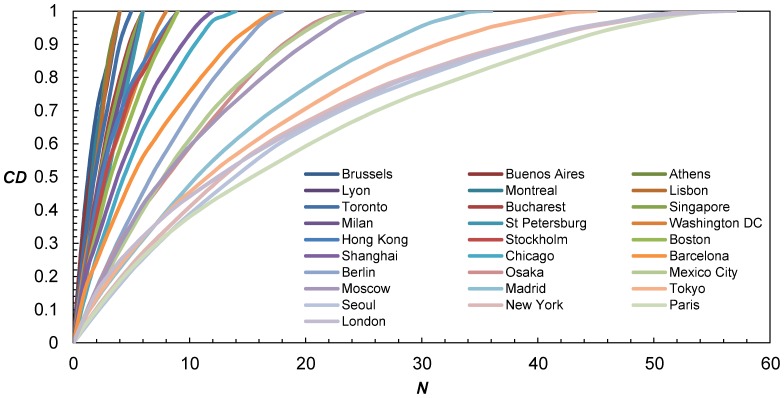
Cumulative distributions (*CD*) of normalized betweenness centrality for 28 metros. Although it can be difficult to pinpoint one specific system, the main message here is that betweenness consistently becomes more distributed with network growth. The absence of a “winner takes all” paradigm is surprising considering it is often the case with other complex network properties (e.g., in scale-free networks).

To further investigate the evolution of betweenness, it is worthwhile to look at the distribution of original and normalized betweenness centrality. Figure 4 shows the cumulative distributions of normalized betweenness for the 28 metros, where the nodes of each system are ordered from largest betweenness to smallest. The figure exhibits many interesting properties, the first being that betweenness consistently becomes more evenly distributed with network size. Indeed, although total betweenness increases as mentioned before, the addition of a new node actually spreads the share of betweenness across all nodes without favoring only a limited number of nodes (i.e. no “winner takes all” paradigm), which can be associated to a process of *democratization*, unlike degree distribution in scale-free networks for instance. As a result, most central nodes in larger networks will retain a lower share of betweenness than most central nodes in smaller networks, which is fairly intuitive and obvious parallels can be made with robustness. This process can be more easily understood through an example. Comparing Athens and London’s metros, it is clear that the share of betweenness of Omonia station (31.25%) in Athens is higher than King’s Cross station’s (8.04%) in London, simply because there are a lot fewer stations in Athens.

Coming back to Lyon, with link EK, the betweenness centrality of each node becomes C (13.5, 0.163), D (19, 0.229), E (9, 0.108), F (20, 0.241), H (21.5, 0.259), and several observations can be made compared to current values. First, the absolute betweenness of all nodes grows regardless of where the addition occurred; this essentially means that the addition of a node does not only benefit a few but it benefits all nodes. That being said, some nodes benefit slightly more than others (e.g., node H takes the lead as the most central node, followed by node F). Second, normalized values shows that shares of betweenness centralities become more distributed in the network. Most nodes “lose” some of their share of betweenness with network growth (e.g., despite increasing its absolute betweenness, the share of node H drops from about 31% to 26%), regardless of where the addition occurs, hence the *democratization*.

This finding has significant impacts. An earlier study [54] showed that few transfer stations tend to retain a certain “monopole” on transferring in metros (i.e. the number of transfers are unevenly distributed in metros). This work here shows that despite this monopole, network expansion invariably leads to a lower share of betweenness for most nodes.

**Figure 5:**
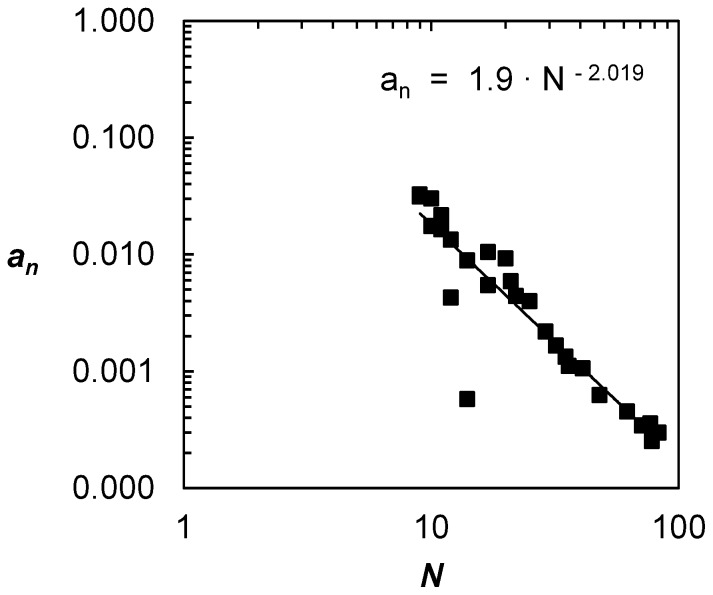
Evolution of quadratic coefficients |*a_n_*| of normalized cumulative distributions with network size.


This phenomenon is particularly interesting and can be estimated numerically by comparing the curves on Figure 4. The relationships have the form of second degree polynomials here as well; i.e. a simple quadratic equation:
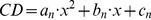
(3)where *CD* is the cumulative distribution, *a_n_*, *b_n_* and *c_n_* are constants, subscript ‘*n*’ stands for normalized, and *x* is the cumulative betweenness. Here again, the quadratic nature of the fit could be due to the planarity of metros. From Table 1, the largest quadratic coefficient |*a_n_*| belongs to Brussels (0.03261, small network) and the smallest belongs to Paris (0.00025, large network). Therefore, concomitant to the democratization process, the value of |*a_n_|* decreases with network size. Moreover, this decrease in *a_n_* actually takes the form of a power law with exponent of approximately 2 (Figure 5) as:

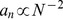
(4)

This relationship is indicative of the evolutionary nature of metro networks in general. Say a metro is being expanded from *N_1_* nodes to *N_2_* nodes, then 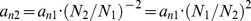; for example, by doubling in size (i.e. 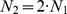), *a_2_* decreases by about a factor of 4 compared to *a_1_* (i.e. 75% smaller). Moreover, the scalar was calculated to be 1.9 (i.e. 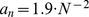). It is uncertain what the impact of this scalar is, but it should be noted that the ratio of links to nodes in metros was found to tend to a value of two with network size [55], and therefore there could be a relation between these two properties.

**Figure 6:**
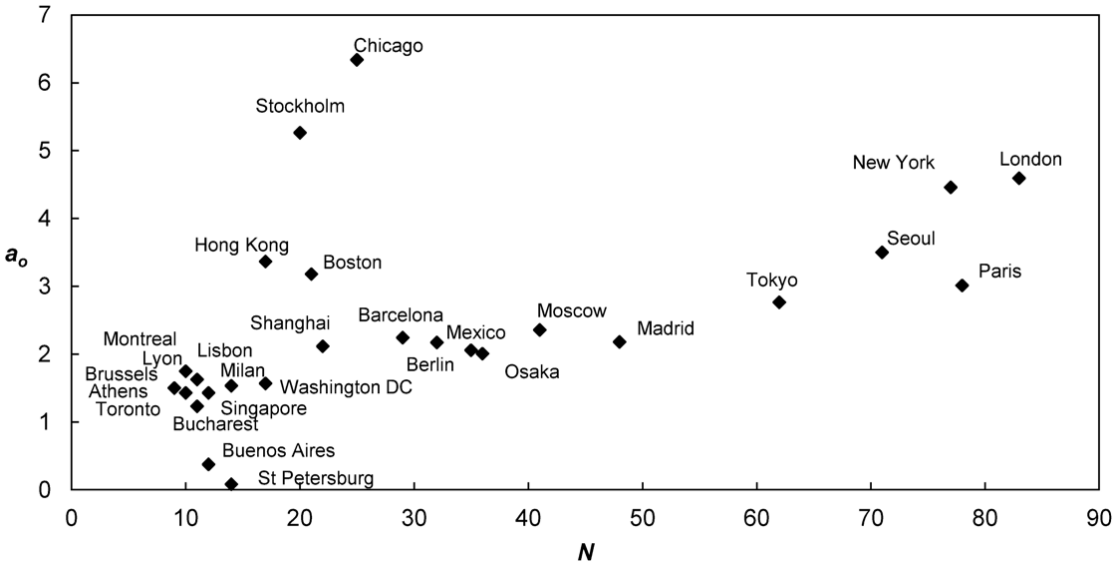
Evolution of quadratic coefficients |*a_o_*| of cumulative distributions with network size. Two clear and distinct regimes can be observed here. The high coefficient of Chicago and Stockholm, whilst being comparatively small, suggests the dominance of a radial feature. On the other hand, the lower coefficient of Paris, considering its size, suggests a dominant grid pattern. New York and London can be seen as hybrids, having fairly high coefficients whilst being large, which is quite intuitive (grid pattern in the center, joined with a radial pattern in the peripheries).

The cumulative distributions of original betweenness values also fit second degree polynomials (not shown here), and their quadratic coefficients |*a_o_*| are displayed in Table 1. Overall, |*a_o_*| has a general tendency to increase with size, which conforms to our expectations (i.e. betweenness increases with size). Nonetheless, two very distinct and surprising regimes can be observed (Figure 6), which are indistinguishable in the normalized version. These regimes are entirely dependent on the structure of the metro and reflect the regional transportation plans that were elaborated by the respective regions. It is therefore possible to relate these values to the overall nature of the metros. For instance, Chicago and Stockholm (famous for linking its satellite towns to the city center) have a high quadratic coefficients whilst being comparatively small, hinting towards a radial structure. In comparison, Paris has a fairly low coefficient whilst having many stations, suggesting a more prominent grid structure. Finally, New York and London can be seen as hybrids, having a high coefficient and being large, which is likely representative of their grid cores and radial lines linking suburban regions to the downtown. This differentiation in structure between metros is interesting, and it may impact factors such as travel patterns and ridership for instance.

**Figure 7:**
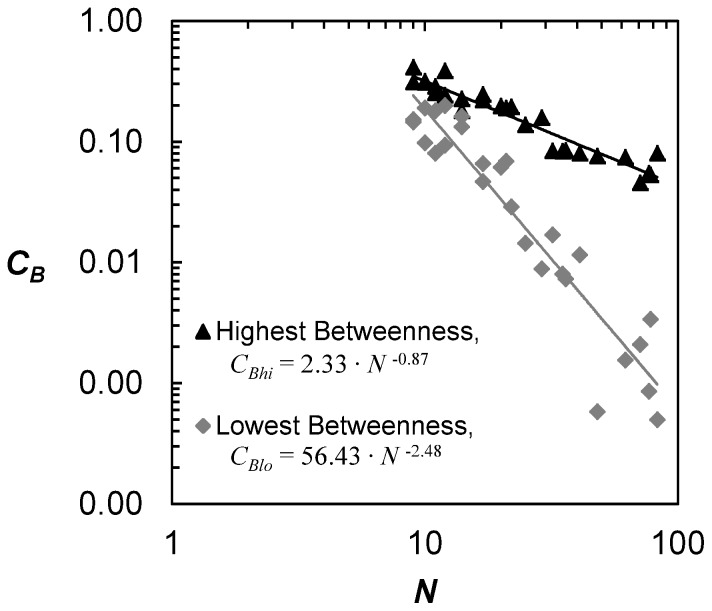
Evolution of highest *C_Bhi_* and lowest non-zero *C_Blo_* betweenness centralities with size. While both centralities fit power law functions, the exponent of highest betweenness is much lower than the lowest betweenness, suggesting that the loss of share in betweenness from most central nodes does not decay as fast as for least central nodes.

*Democratization*, however, does not mean ‘equalization’. Indeed, despite an overall share loss, some nodes benefit slightly more than others, which are most often the nodes directly connected to the new stations/lines. As a result, it is worthwhile to further investigate the effect of size on three specific values: highest betweenness centrality *C_Bhi_* (i.e. node having the highest value of betweenness), average betweenness *C_Bav_* and lowest non-zero betweenness *C_Blo_*. Normalized values have to be used here to be able to compare metros with one another. Average betweenness centrality is defined as 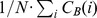, dividing it by the sum of centralities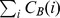 to normalize it results in the inverse function 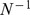, thus an exponent of 1. As shown in Figure 7, highest betweenness centralities share a strong power law relationship with network size, with an exponent of 0.87. A similar relationship exists with lowest betweenness with exponent 2.48 (Table 2). The difference in exponents suggests that the share of the node with highest betweenness decreases slower than the average, and the share of the node with lowest betweenness decreases faster than the average. In other words, nodes with highest betweenness centralities remain comparatively more central in the system, and despite a share loss, they benefit more than the average. Nodes with lowest betweenness centralities on the other hand, are proportionally less central with network size, and their betweenness centralities decrease at a faster rate than the average. This phenomenon is all the more interesting that most central nodes are not only the nodes that possess many transfers, but also those are simply topologically well located in the network (i.e. at the center), as we are about to see in the next section.

**Table 2:** Node betweenness and power law exponents.

NodeBetweenness	Power LawExponent
*C_Bhi_*	0.87
*C_Bav_*	1.00
*C_Blo_*	2.48

Overall, the properties uncovered are insightful about the nature of metros, and a similar study on other networks would be valuable, notably on other urban infrastructure networks to identify potential synergies [56]. In the next section, the analysis is brought one step further by locating and studying the most central nodes in larger networks, which can be critical to relieve some of the pressure from current systems by redistributing passengers.

### Individual Systems

As a practical application, betweenness centrality can be used to determine which stations are topologically more central in the system. At the moment, transit planners assess network centrality either geographically (i.e. stations in the city center), by identifying major transfer hubs (related to degree centrality), or at best by looking at platform counts (i.e. actually usage of the stations). Using the concept of betweenness centrality therefore offers clear benefits to identify stations that are naturally (or topologically) central. In particular, and as previously mentioned, locating most central stations can be of significant help to redistribute passengers to stations and lines that experience lower volumes.

As opposed to the previous section, the centrality of each station is calculated here. Table 3 contains the original and normalized centrality values of the five stations with highest betweenness for all metros having at least 20 nodes. To illustrate the value of this method, the bulk of this section consists in going through several familiar systems and discussing the opportunities. These systems are: London, Paris, Madrid, Chicago, and New York.

**Table 3:** Five most central stations and their betweenness centralities for metros with *N* ≥20.

**Cities**		
Stations	***C_B_***	***C'_B_***
**Stockholm**		
T-Centralen	113	0.198
Slussen	103	0.180
Gamla stan	90	0.157
Fridhemsplan	63	0.110
Gullmarsplan	63	0.110
**Boston**		
Park St	102	0.190
Copley	79	0.147
Downtown Crossing	75	0.139
Gov't Center	59	0.110
Kenmore	54	0.100
**Shanghai**		
People's Square	93	0.194
Century Avenue	53	0.111
Zhongshan Park	53	0.111
Shanghai Indoor stadium	50	0.105
Shanghai South Railway	41	0.086
**Chicago**		
Roosevelt	221	0.138
Fullerton	193	0.121
Washington-Blue	168	0.105
Belmont	166	0.104
Jackson	143	0.089
**Barcelona**		
Diagonal Provenca	163	0.159
Verdaguer	90	0.087
Sants Estacio	89	0.086
Maragall	84	0.081
Passeig de Gracia	82	0.080
**Berlin**		
Leopold platz	110	0.084
Stadtmitte	106	0.081
Alexander platz	100	0.077
Zoologischer garten	99	0.075
Bayerischer platz	91	0.069
**Mexico City**		
Chabacano	129	0.084
Tacubaya	105	0.068
La Raza	99	0.064
Centro Medico	98	0.064
Consulado	95	0.061
**Osaka**		
Hommachi	153	0.085
Tanimachi9-chome	141	0.078
Sakaisuji-Hommachi	135	0.075
Nippombashi	119	0.066
Namba	97	0.054
**Moscow**		
Aleksandrovski Sad	177	0.080
Kurskaya	177	0.080
Pushkinskaya	153	0.069
Marksistskaya	149	0.067
Okhotny Ryad	136	0.061
**Madrid**		
Avda de America	265	0.076
Sol	190	0.054
Plaza de Espana	173	0.050
Pacifico	156	0.045
Alonso Martinez	154	0.044
**Tokyo**		
Otemahi	453	0.037
Hlbiya	370	0.030
Shinjuku	309	0.025
Akasaka-mitsuke	296	0.024
Kasuga	257	0.021
**Seoul**		
Express Bus terminal	468	0.046
Gunja	466	0.046
Daerim	439	0.043
Konkuk Univ	424	0.041
Isu	422	0.041
**New York City**		
Broadway Junction	683	0.055
Metropolitan Av Lorimer	618	0.049
59 st Columbus circle	570	0.046
42 st Times Sqr	541	0.043
Lexington Av 59 st	521	0.042
**Paris**		
Republique	631	0.053
Chatelet les Halles	541	0.045
Gare de l Est	531	0.045
Gare Saint-Lazarre	503	0.042
Madeleine	461	0.039
**London**		
Kings Cross St Pancras	1240	0.080
Baker Street	1142	0.074
Bank	755	0.049
Moorgate	685	0.044
Liverpool Street	578	0.037

London is the largest system considered in this analysis. The station with highest betweenness is Kings Cross St Pancras, which is also a major train station (i.e. likely to be congested) and is geographically centrally located. The second station is Baker St., which is located fairly closely to the former and can serve as a hub for the north-western part of London. Note that Baker St. station actually has more connections (i.e. higher degree centrality) than Kings Cross St Pancras, but unlike the latter, it does not have a direct access to the Piccadilly and Victoria lines, which are two major diametrical lines with abundant transfer stations. The third station is Bank, and the fourth is Moorgate. Both these stations host the Northern line, which is also shared with Kings Cross St Pancras; hence having a particular potential to redistribute passengers (their location in the system seems to be the decisive factor that gives them high betweenness). The fifth station is Liverpool Street, which is also a train station. It is interesting to observe that none of these stations are located in the central area of London (i.e. inside the area delimited by the Circle line) such as Piccadilly Circus or Oxford Circus. The first station located within this area is Holborn station, coming in 12^th^, followed by Green Park in 14^th^ position, both with betweenness centralities that are roughly a third of Kings Cross St Pancras.

The Paris metro is also very large in size and is famous for being ubiquitous in the city. The station with highest betweenness is République. The République station is the main hub to link the north-eastern part of Paris to the rest of the city. The Bastille station is also very important to further link eastern Paris with the rest of the city, but it actually comes in 11^th^ place, having less than half the betweenness of République. The second station is Châtelet Les Halles, which is one of the main hubs in the Paris system and a very busy station. It is surprising to see that Châtelet Les Halles comes in second place (and by a significant margin), considering it is geographically more central than République. They both host the same number of lines, but somehow, the lines hosted by République grant it better betweenness. The third and fourth stations, Gare de l’Est and Gare Saint-Lazarre, are both train stations that are likely to be busy; note, however, that despite having a lower number of connections (i.e. degree centrality), Gare de l’Est is slightly more central, likely thanks to its direction connection to République. Unexpectedly, the fifth station is Madeleine, which is well located in the center of the city, but it has much fewer connections than other stations such as Montparnasse Bienvenüe, which comes in 7^th^ place. Madeleine is not known to be a major transfer point, it could therefore have some potential to attract passengers.

The Madrid metro is known as a success story in the transit community, since it has doubled in length in ten years. The station with highest betweenness is Avda de America, located in the north-eastern part of the city, acting a transfer hub. It is actually also the station with the most connections (i.e. highest degree centrality). The second station is Sol, located in the famous Puerta del Sol, and the third is Plaza de Espana, situated close to the Royal Palace. Surprisingly, the fourth station is Pacifico, which is located in the south-eastern part of the city. Its high betweenness seems heavily reliant on the fact it is directly connected with Sol, thus presenting some potential to relieve pressure from it, but also because it hosts the Circular line. Finally, the fifth station is Alonso Martinez, which acts as a major transfer point. Despite having mostly diametrical lines, the Madrid metro seems to possess a grid structure, which particularly allows for multiple transfer stations. This grid property is further supported by a comparatively low |*a_o_*| in Figure 6.

Contrary to Madrid, the Chicago metro has a strong radial structure, which requires that most trips go through the city center. Its station with highest betweenness is Roosevelt, which is located at the southern tip of the loop. The second station is Fullerton, which appears to carry an equivalent function to Roosevelt station, but north of the loop. The third station is Washington-Blue that is located in the center of the loop. Surprisingly, the fourth station is Belmont; it is situated north of Fullerton station and it therefore seems to have some potential. The fifth station is Jackson, which is close to Washington-Blue. It is interesting to notice that none of these stations are part of the elevated system present in the loop. In fact, only the sixth station, Clark station, is part of the elevated loop system.

The New York subway is also a significantly large metro that is partially shaped by the geography of the region; i.e. Manhattan’s topography favors North-South lines. The station with highest betweenness is Broadway Junction located in Brooklyn. It is followed by Metropolitan Av Lorimer St that is also in Brooklyn. This is fairly surprising considering Manhattan is the logical center of the New York City transit system. These results essentially mean that the natural center of the New York City subway is in fact located in Brooklyn. Consequently, it may be desirable to direct future expansion of the network to shift centrality to Manhattan, as may be the case with the construction of the new Second Avenue line currently scheduled for 2016. The three other stations are located in Midtown Manhattan, they are: Columbus Circle, Times Square, and Lexington Ave at 59st. Because of the geography of New York City, taking a topological framework to future planning might be of significant help to control the flow of passengers within the system.

Finally, it should be noted that this approach does not take the presence of other modes into consideration. For instance, a station might be strongly affected because it offers a transfer with a regional rail system, or it is linked with a strong feeder services from a light rail, bus rapid transit or even a conventional bus system. Similarly, other stations may be overrepresented, which is particularly true of main junctions for multiple branches (e.g., Harrow-on-the-Hill station in London, or Copley station in Boston). As a result, creating artificial branches (or alternatively removing actual branches) in the adjacency matrices may be desirable to simulate these exogenous factors. By creating or removing nodes, weights are essentially applied, therefore increasing (or decreasing a node’s betweenness). Although this solution may not be optimal, it is pragmatic and easy way to perform a relatively simple analysis and compare different scenarios.

## Discussion

If the cities are to solve the serious challenges the earth is facing, public transport systems are likely to take an increasingly important role to provide mobility to the growing urban population. Current transit systems, however, are undergoing a lot of stress and they are not capable to accommodate the increasing demand. Adopting a complex network approach to develop new tools and technics for transit planners can therefore be beneficial.

This paper dealt with one of the most important aspect of transit systems: network centrality. In our case, centrality was assessed by using betweenness centrality. Betweenness measures the importance of a node as a transfer point to join pairs of nodes, and its relevance to transit is self-evident. It was applied to 28 metros in the world that range all sizes. More specifically, the main goal of the paper was to identify global trends in the first place, followed by a more detailed analysis of individual stations in several systems.

The methodology applied to study metros was first introduced, where only transfer stations and termini were considered. The concept of betweenness itself was then defined as the sum of the ratios of all shortest-paths going through a particular node and the total number of shortest-paths.

By comparing the systems at the global level, clear patterns were found that had surprisingly strong statistical significance despite the fact metros were built independently of each other. First of all, although betweenness increases with network size by definition, using normalized cumulative distributions showed that the share of each node consistently decreases (i.e. no “winner takes all”), which was referred to as a process of *democratization*. Moreover, these distributions had second degree polynomial fits, whose quadratic coefficients decrease with network size following a power law of exponent 2. Looking at the original (non-normalized) cumulative distributions showed similar second degree polynomials. In this instance, however, the quadratic coefficients exposed two distinct regimes in the nature of metros, enabling us to differentiate between radial vs. grid structures. Finally, a further detailed analysis of nodes with highest, average, and lowest betweenness revealed that they all decrease in a power law fashion with network size. Nonetheless, the share of nodes with highest betweenness decreases slower than average (0.87 vs. 1), while the share of nodes with lowest betweenness decreases faster than the average (2.48 vs. 1).

Subsequently, effort was concentrated on looking at systems individually by locating the five most central stations of all metros having more than 20 nodes. In particular, five systems were examined more closely and specific stations were identified that could be used to redistribute passengers in the network (thus relieving stress from overcrowded stations). One finding was that stations located in the center of the network tend to have higher betweenness centralities, simply because they are connected to other stations with high betweenness. Taking considerations of centrality into account in the planning process can be valuable, notably to control centrality (e.g., back to Manhattan), and therefore better distribute the flows of passengers.

Overall, centrality is an important notion in network science and it is at the core of public transport. Better understanding the topology of transit systems can be valuable and helpful for scientists, planners and engineers. Adopting a similar framework to study urban infrastructure systems in general could be very promising as well, especially to compare the network topologies of the different systems (e.g., how does the topology of the electric grid compare with the water/wastewater system, etc.). Much work therefore remains to be done.
